# Nonlinear interferometers with correlated photons: toward spectroscopy and imaging with quantum light

**DOI:** 10.1038/s41377-020-00363-y

**Published:** 2020-07-17

**Authors:** Konstantin E. Dorfman

**Affiliations:** State Key Laboratory of Precision Spectroscopy, East China Normal University, Shanghai, 200241 China

**Keywords:** Imaging and sensing, Quantum optics

## Abstract

A nonlinear optical interferometer based on crystal superlattices has been demonstrated for the first time in a cascade of up to five crystals. The enhanced sensitivity due to quantum interference and correlations makes it a promising tool for sensing, imaging, and spectroscopy.

The idea of detecting low concentrations of atoms and molecules down to a few tens of particles per million is a fascinating task. The infrared (IR)-based optical sensors required to detect small changes in molecular internal motion, which constitute fingerprints for the sensing and identification of the chemical constituents, are not widely available due to limitations on the optical materials in the IR region^1^. Interferometric methods based on nonlinear optical processes that are based on nonlinear crystals^2^, gas cells^3^, fiberized networks^4^, and nonlinear waveguides^5^ may solve these limitations in part. Typically, these methods rely on the ability to detect small changes in the interference pattern, such as a shift in the fringes or change in the fringe visibility.

One of the possible solutions based on crystal superlattices was proposed by Klyshko^6^. A setup with *N* nonlinear elements separated by linear gaps provides a higher spectral resolution when the number of crystals is increased. Each crystal constitutes a parametric process, where one higher energy (pump) photon generates a pair comprising a lower-energy signal and an idler photon that have quantum correlations. While the theoretical idea guides the experimental demonstration, it poses the following challenges that have to be addressed. First, the quantum correlations between the signal and idler photons have to be preserved across multiple nonlinear elements. Furthermore, the increased number of nonlinear wave mixing processes requires a higher degree of alignment and stability, which is difficult to achieve.

In a recent publication^7^, Paterova and Krivitsky demonstrated experimentally for the first time an efficient nonlinear interferometer based on a superlattice consisting of a sequence of nonlinear crystals (Fig. 1a) pumped by a single coherent laser. The robust and stable alignment resulted in a well-controlled interference pattern in the frequency-angular spectrum with high visibility for five crystals compared with the case of two crystals (Fig. 1b). A proof-of-concept gas sensing experiment showed enhanced sensitivity. Compared with previous reports, the novel system presents several advances. First, the previous works typically addressed two nonlinear elements, while the new setup demonstrates the use of up to five nonlinear elements and is not limited to this number. Second, the novel setup allows flexibility in the realization of alternative crystal configurations by enabling the sizes of the crystals and gaps between them to be adjusted, suggesting novel ways to carry out quantum state engineering. Third, the novel setup can be extended to other optical platforms, such as integrated photonic circuits and fiber platforms. Finally, the setup can be applied in the many-photon regime, which can potentially improve the collection time and feasibility of the scalable device. Overall, these capabilities suggest an attractive development in the area of high-performance devices for sensing, metrology, spectroscopy, imaging, and quantum state engineering.

**Fig. 1:**
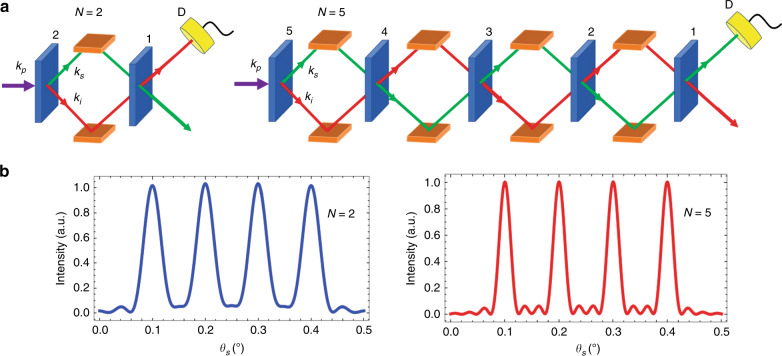
Nonlinear interferometer based on crystal superlattices. **a** Schematic of the nonlinear interferometer with a crystal superlattice. *N* = 2 (left) and *N* = 5 (right) identical nonlinear crystals, separated by equal gaps, are coherently pumped by a pump laser with momentum k_p_, which generates a pair of correlated signal k_s_ (green arrow) and idler k_i_ (red arrow) photons, which are then redirected to the next crystal. The intensity of the signal photons is measured in the experiment by detector D. **b** Cross sections of the interference fringes for the nonlinear interferometer with two (left) and five (right) crystals
